# Does Trauma-Informed Care Have a Place in Audiology? A Review and Practical Suggestions

**DOI:** 10.3390/audiolres13060080

**Published:** 2023-11-10

**Authors:** Shade Avery Kirjava, Jennifer Phelan

**Affiliations:** 1 Department of Health, Society, and Behavior, University of California, Irvine, CA 92617, USA; 2 Department of Communication Sciences and Disorders, University of South Dakota, Vermillion, SD 57069, USA; jennifer.phelan@usd.edu

**Keywords:** trauma-informed care, adverse childhood experiences, pediatrics, veterans, vestibular testing

## Abstract

Background. Trauma from adverse childhood experiences (ACEs) and serious traumatic events in adulthood is a significantly prevalent concern for public-health-hearing healthcare professionals. The pediatric and geriatric populations that audiologists often work with have been shown to be at an increased risk of experiencing traumatic events. Childhood and adult trauma can significantly impact the hearing and vestibular testing and treatment of these patients. Methods. This narrative review article discusses trauma-informed care (TIC) strategies that audiologists can use to recognize and respond to trauma in patients and prevent retraumatizing patients during their encounters in audiology clinics. Conclusions. This article will provide an overview of TIC and direct the reader to resources for their continued learning. Practical guidance on implementing trauma-informed practices in clinical audiology are also provided.

## 1. Introduction

Significantly negative medical, interpersonal, and violent events can be traumatic to the individuals experiencing them. Trauma has been defined as “individual trauma as an event or circumstance resulting in physical harm, emotional harm, and/or life-threatening harm” and can be caused by natural disasters, interpersonal violence, and traumatic medical interventions [1,2]. Nearly two-thirds of people have a history of traumatic events during childhood, called adverse childhood experiences (ACEs), or traumatic events during adulthood [2,3,4]. When an individual experiences traumatic events that cause persistent stress, they may experience changes in the epigenetic expression of how an individual’s genes function, neuroplasticity, and changes to hearing and balance including tinnitus, auditory processing changes, and a reduced ability to participate in audiologic testing and treatment [3,4,5,6]. Though psychological trauma receives less attention than other types of traumas in clinical audiology, the high prevalence of traumatic events, the legal requirement in which audiologists are mandated reporters, and the way that trauma can disrupt audiologic testing and treatment means that all audiologists providing direct patient care should be aware of trauma and its effects.

**Figure 1:**
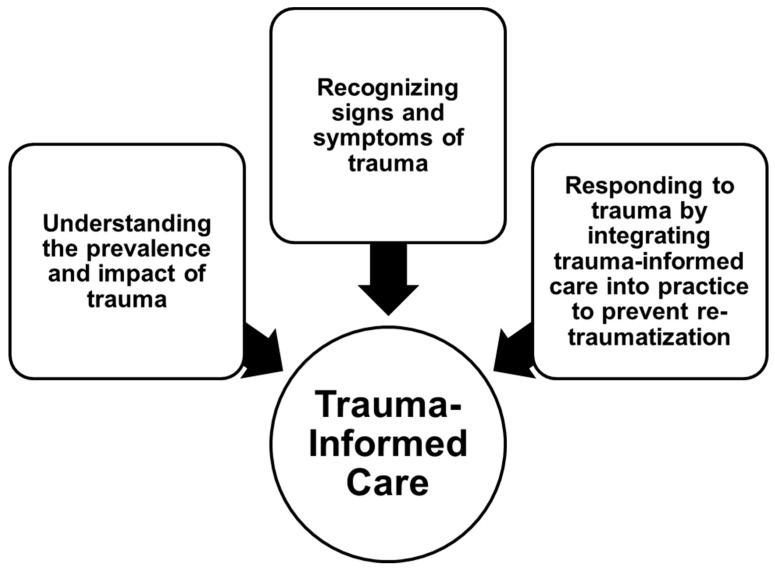
Trauma-informed care model.

Accounting for trauma in audiology patients is important to provide these individuals with high-quality, person-centered care. Childhood trauma has received significant attention in other allied health disciplines, so in recent years all healthcare providers working with patients have been encouraged to become competent in trauma-informed care (TIC) and adult-specific strategies [6]. TIC includes discipline-specific strategies to identify and assess trauma, and prevent a pediatric patient from being re-traumatized by the healthcare services they are receiving, as in Figure 1 [6].

Few audiology-specific resources in the literature or from professional organizations are available for clinicians to become trauma-informed or to learn about the effects of trauma on their practice. Understanding TIC and adult-specific strategies for patients who have a history of trauma may improve an audiologist’s provider–patient relationship, increase a patient’s compliance with testing and treatment, and prepare a clinical audiologist to work effectively and efficiently with patients who have a history of trauma. This article summarizes the available literature on trauma-informed care as it applies to clinical audiology and provides concrete suggestions for clinicians wishing to provide more trauma-informed services.

## 2. Trauma

### 2.1. Trauma in Childhood

Many sources of trauma have been identified in the literature. The most common source of trauma in children is within the home. Caregiver neglect and abuse are the most common ACEs, though caregiver substance use, mental illness, or death are also possible sources of ACEs [4,6,7]. In addition, domestic violence and sudden or frequent relocation have also been shown to be sources of trauma [4,7]. Though trauma outside of the home is less common, research has also shown that natural disasters, community violence, terrorism, and discrimination are also ACEs [6,7].

Though little research on ACEs in audiology clinics is available, medical trauma is a pertinent source of ACEs for children with chronic health conditions. With millions of children being hospitalized or interacting with the healthcare system every year, the injuries, conditions, and procedures that these children experience have the potential to be traumatic, particularly if children experience repeated or subjectively invasive procedures or experience peer teasing from them [4,6,7,8]. Trauma from other medical settings can interfere with effective hearing testing and treatment at audiology clinics.

Some populations of children are particularly vulnerable to trauma. These groups are not mutually exclusive, and multiple traumatic events from different sources are possible. Populations that are at a high risk of trauma include historically marginalized and underrepresented groups such as racial, ethnic, or religious groups with current or historic trauma, LGBTQ+ children, and neurodivergent children [6,9]. Children in foster care or, poverty, who are refugees or immigrants, and children in military families have all also been highlighted as high risk [6,9]. In addition, children with complex or chronic health conditions such as hearing loss or balance disorders that include medical interventions have also been found to be at a high risk of medical trauma due to the repeated, invasive healthcare encounters with healthcare providers [9].

About 64% of the population may have been affected by at least one ACE, with 60% of children possibly having an ACE in the last year—nearly two-thirds of patients seen in an audiology clinic may be affected by these events [4,6,7]. If one ACE is present additional ACEs are more likely to also be present, with about 6% of individuals having four or more ACEs [4,10]. Safe, stable, nurturing adult relationships can provide a protective buffering effect to prevent these ACEs from causing significant symptoms.

The COVID-19 pandemic has the potential to be an ACE for the vast majority of children through the loss of support and resources for vulnerable children, caregivers or family members dying or becoming unemployed, and them being socially isolated from others [11]. COVID-19 also caused schools and daycares that can insulate children from home trauma to close and reduce the opportunities for teachers and other staff to detect and report maltreatment, so audiologists may encounter more patients with ACEs and previously undiagnosed trauma from COVID-19 and related societal disruption in the coming years [11].

### 2.2. Trauma in Adulthood

Though TIC was originally concerned with children to young adults 21-years-old, many adult and geriatric audiology patients also experience and struggle with trauma, often from the same sources as ACEs, such as the COVID-19 pandemic. Serious traumatic events in childhood or adulthood that are witnessed or experienced can result in trauma in adults that affects how these individuals interact with audiologists [3]. Many diverse experiences can cause trauma responses of various strengths, from natural disasters, like tornadoes to accidental and intentional acts, like robberies or shootings [8,12]. For a review of the types of events that often cause trauma, see [2].

Trauma is particularly common in some subpopulations that are also often seen in audiology clinics for hearing and balance disorders, such as veterans who have been reported to have rates of post-traumatic stress disorder (PTSD), as high as 17.1% for some populations of veterans [13]. In addition, people later in life engage with memories to make meaning of their life, which may cause PTSD symptom reemergence in older adults who are also more likely to visit audiology clinics for hearing and balance disorders that also become more common with advanced age [5,14]. These factors potentially cause a relatively higher comorbidity of PTSD and hearing or balance disorders in the patients in an audiology clinic compared to other disciplines.

Tinnitus is often comorbid with PTSD. Tinnitus has been shown to begin after traumatic events, to potentially trigger intrusive traumatic memories, and to be worsened by anxiety from PTSD due to the limbic system involvement in both PTSD and tinnitus [3,5]. Among people with both PTSD and tinnitus, tinnitus may be subjectively louder and more bothersome, remain bothersome for an extended period, and more often cause difficulty sleeping or concentrating [5].

Hearing loss, PTSD, and substance use are also often comorbid. This is partly because disassociation triggered by substance use is used by some individuals to self-treat PTSD [15]. In addition, hearing loss is a barrier preventing access to mental health and addiction services, and communication barriers from hearing loss inhibit the learning of healthy coping behaviors causing people with PTSD and hearing loss to be more likely to learn coping strategies from peers that may not be healthy or effective, such as substance use [15].

### 2.3. Effects of Trauma

Stress can be beneficial to an individual, but stress becomes toxic if stressful factors outweigh protective factors, causing the chronic activation of an individual’s stress responses [4], but, stress from traumatic events can develop into traumatic toxic stress, the frequent or prolonged activation of physiologic stress responses [6]. Toxic stress causes more significant changes when the trauma occurs at a younger age and when the trauma is more severe or prolonged [6]. Traumatic toxic stress causing the extended activation of stress responses has been shown to cause significant changes in people who experience the stress. This toxic stress causes changes to an individual’s body, from epigenetic changes to DNA that lasts a lifetime, to cellular and organ-level changes, to functional differences in physical, emotional, developmental, and behavioral health across the lifespan [4,6,16].

Trauma in childhood or adulthood can result in traumatic toxic stress, which can cause maladaptive neuroplastic changes to an individual’s CNS resulting in poor regulation of sensory input, poor habituation to sound, poorer auditory recall, hyperacusis, auditory processing dysfunction, and increased distress from tinnitus [3]. Other more general symptoms of trauma include depression, anxiety, substance use, headache, and other symptoms associated with hearing and balance disorders that can complicate the accurate assessment of these patient’s hearing and vestibular function [8,17].

Audiologists must be conscious of traumatic events in their patients because ACEs and traumatic events experienced as adults have been linked to chronic health conditions, including stroke, cancer, autoimmune disorders, anxiety, and depression, all of which are associated with hearing and vestibular health [4]. In addition, toxic stress can decrease employment and healthcare access and increase risky health behaviors, such as substance abuse, all of which inhibit patients from accessing audiologic healthcare and following their audiologic treatment plan [4,8].

## 3. Trauma-Informed Care

TIC is recognizing and responding to the impact of traumatic stress on the patients, caregivers, and healthcare providers in a healthcare setting [18]. Though TIC was originally focused on newborns up to 21-year-old young adults, many of the principles are also beneficial for adults who have a history of trauma [9]. Advocates and researchers have called for all clinicians providing direct patient care to be trauma-informed in discipline-specific strategies [9].

TIC involves identifying and appropriately responding to trauma in patients while avoiding retraumatizing a patient with the clinical services they are receiving [2,6]. TIC strategies should be incorporated into the policies and procedures to prevent re-traumatization, such as office staff being trained to refer to patients with their preferred name and pronouns for gender diverse patients [4]. TIC also considers the detrimental effects of secondary traumatic stress on caregivers and healthcare workers who experience stress from hearing about trauma from people who have experienced trauma [6]. Caregivers and healthcare providers who display symptoms of toxic stress may be referred for supportive mental health services.

Accounting for the effects of trauma is essential to accurately diagnose, refer, and treat individuals with a history of trauma [6]. Though little research exists on TIC in audiology, in other fields, neglecting to account for the impact of trauma and distress during clinical testing may result in more difficulty obtaining reliable results, high patient no-show rates, patients dropping out of testing before completion, poorer patient experiences, and less income due to the increased time that must be spent managing patients in distress [19].

Research suggests that childhood trauma is relevant to clinical audiology and is important for clinicians to consider in the provision of hearing healthcare. Links between trauma and audiology include, for example, that childhood trauma has been linked to adult anxiety, which is associated with several balance disorders. In addition, examples of trauma from being in foster care have been linked to auditory processing changes in children [6,17].

Readiness to work with people who have a history of trauma should include focusing on a patient’s strengths and competencies, not on problems or deficits [10]. For example, a clinician could consider emphasizing strategies to promote effective communication in a patient’s activities of daily living instead of extensively discussing the frequencies and speech sounds that a patient cannot hear because of their hearing loss. Gradually easing patients into clinical testing, acting predictable, and empowering an individual to direct their own care are all trauma-informed strategies to prevent retraumatizing patients during clinical encounters [8,20]. In many ways practicing using strategies that are trauma-informed will benefit all patients.

Clinicians working with children and families can also implement strategies to make the patient-provider power dynamic more palatable. Establishing routine, allowing children to make choices that the clinician can accept, respecting a child’s boundaries, and reducing child-specific triggers can all improve the interactions between a child with a history of trauma, and a clinician [20,21]. These strategies also support the clinician–parent relationship. Having parents as partners in the process of working with their children often makes the testing situation less bothersome for both the patient and their guardian.

### Preparing Patients for Challenging Testing

Warning patients about potentially distressing situations that they may experience may seem intuitively beneficial, but these warnings have not been shown to reduce distress from testing. Telling patients that they may feel dizzy and nauseous and may vomit during vestibular testing, for example, makes all of those outcomes more likely [22]. Audiology patients should be acquainted with testing and discuss the possible uncomfortable outcomes carefully to avoid priming patients to believe that these outcomes are guaranteed.

This is especially important when working with children and their families. In this context, it may not be the child’s history of trauma, but the parent’s past that also needs to be considered. When conducting an evaluation, such as otoacoustic emissions, a child may cry or show signs of distress which can in turn stress their guardian. In this situation, it may be best to give a brief summary of the testing in general terms so they are able to comfort the child knowing the testing is not causing pain. Using general terms, such as “tip” versus “probe”, is an example of using language that could be less agitating to the individuals who may be needed to support the patient.

Content warnings, also called trigger warnings, have been proposed as a tool to reduce distress in response to challenging situations, such as claustrophobic audiologic testing, among people exposed to trauma, though the available evidence suggests that trigger warnings are not an effective tool to reduce distress among people with a history of trauma. Though these warnings may intuitively seem beneficial to reduce the toxic stress experienced by people with a history of trauma, the current body of evidence does not support their use in educational or clinical settings.

Triggers are automatic reflexes for some people who have experienced trauma that cause intense fear or horror and other symptoms, such as experiencing intrusive thoughts related to the trauma. These triggers are stronger in people with trauma, and less strong in people with specific phobias, such as claustrophobia. Trigger warnings are intended to prompt someone that potentially distressing content, such as loud, sudden sounds in acoustic reflex testing, will soon be encountered. Today they are present in many settings, including healthcare higher education where they are used to prepare healthcare graduate students for potentially distressing course content [23]. Proponents of trigger warnings suppose that they provide a protective effect by letting an individual that might be triggered by trauma call to mind helpful coping mechanisms before they engage with the distressing material to reduce the intrusive thoughts and other PTSD symptoms that can be caused by encountering triggering material.

Trigger warnings have only been seriously studied in recent years and have not been investigated in audiology. What research exists indicates that, contrary to the intended effect, trigger warnings provide no protective effect from distress or that trigger warnings slightly increase anxiety from anticipating the distressing content, particularly for people with lower SES who are less able to access mental health services and learn effective coping strategies that they can call to mind [23,24,25]. Researchers have also found that trigger warnings exacerbate negative emotional reactions to distressing material [24], may reinforce how central trauma is to someone’s identity, inhibiting an individual from processing and moving on from trauma, and may encourage temporary avoidance, which maintains or worsens long-term PTSD symptoms [23]. Content and trigger warnings do not appear to be an effective trauma-informed strategy for clinical audiology.

## 4. Becoming Trauma-Informed

Despite the high rate of adverse childhood experiences and adult trauma in the patients that audiologists often see, trauma-informed care has received little attention in audiology. Many clinicians within allied health professions are not trained in TIC or are unwilling to implement TIC in their practice, possibly because of their own experiences of childhood trauma [26]. Clinicians’ own histories need to be considered when any discussion of trauma is raised. This will allow them to acknowledge and work on aspects of their own past that may limit their ability to provide compassionate care. In addition, all clinicians may benefit from supportive counseling to avoid compassion fatigue and burnout from secondary traumatic stress. Mental health services are valuable to learn effective coping strategies regardless of the clinicians history of trauma or workplace setting [6]. Providing healthcare services to other individuals can be taxing on the provider. It is important that trauma-informed care start with self-care.

Many training opportunities that increase someone’s confidence and perceived ability to be trauma-informed exist in graduate and continuing education [7,26]. TIC training should be discipline-specific, but as of the time of writing, no audiology-specific resources or trainings are readily available. Research has shown the effectiveness of TIC training in other allied health professions, including physical therapy, occupational therapy, and speech-language pathology [9,16]. A good place to start would be for audiology students and clinicians to review Marsac et al., 2016 [7], for a discussion of TIC resources from the Center for Pediatric Traumatic Stress, the American Academy of Pediatrics, and other organizations. While this article can present information and gives a clinician somewhere to start, a single article is not sufficient to become trauma-informed. Readers are encouraged to continue to engage with TIC information in their continued learning. As members of an allied health profession, audiology clinicians can go to their professional organizations and others who provide continuing education to ask for TIC teaching that is audiology-specific.

Special attention should be given to audiology undergraduate and graduate students when training them on TIC. Because of the high prevalence of childhood trauma among students and because many students have mood, anxiety, or substance-use disorders, student clinicians may experience distress when learning about trauma and TIC [10,24]. Students learning about trauma may benefit from training on tolerating their trauma-related triggers and cope effectively with the stress that discussing trauma can elicit [27]. Universities are uniquely prepared to provide this training as they also have resources students can tap into for personal support.

Trauma-informed providers should be aware of the prevalence and effects of trauma, be ready to work with people who have a history of trauma, be able to detect a history of trauma in their patients, manage these patients, and integrate trauma-informed strategies into their clinical practice setting. Awareness of the nature and prevalence of the complex interacting factors affecting trauma is essential to being trauma-informed [6,7,16]. Simply reading an article is not enough for a clinician to provide TIC. This person may be aware of the effects of trauma, but specific care based on that trauma takes training.

Audiologists may be the first professionals to detect trauma, though since most minors seeing audiologists are also receiving care from other healthcare professionals, audiologists are not solely responsible for detecting and assessing trauma in their patients [6]. Audiologists must, however, be willing and able to detect and report trauma, as most states require audiologists to be mandated reporters for both children and the elderly [14,28].

The American Academy of Pediatrics recommends using screening instruments, like the Psychosocial Assessment Tool, for children with a possible history of trauma to check for childhood medical trauma in children with a significant history of medical involvement [10]. Adult questionnaires, like the PHQ-9, can be included in standard intake and history-taking or given to patients who are at a higher risk of trauma [29]. Comprehensive overviews of screening tools and their use are available in the literature; clinicians may choose a screening tool based on the ages they see in the clinic, the availability of parent or guardian perspectives for adult patients, and the type of trauma most often seen in their patient population [30,31]. Trauma should be periodically rescreened at follow-ups, particularly for ongoing situations that may be traumatic, such as unstable housing [6]. Adding these screening tools to the toolbox of resources audiologists have will enable clinicians to provide the best care for their patients.

In addition to formal screening, a clinician should also look for functional symptoms of trauma including altered behavior or neurobehavioral symptoms, like poorer developmental milestones. A rapport with patients should be built before discussing trauma as discussing trauma can be significantly distressing. When discussing trauma with a patient a clinician can use active listening, open-ended questions and nonthreatening body language to make the patient or family member more comfortable in the discussion [6]. This is a tangible example of the importance of audiologists as a part of a patient’s healthcare team.

An audiologist’s management of a patient with trauma may include referral to mental health professionals, other members of a patient’s care team, or other supportive resources, such as local food banks [6]. Among hearing aid users, a manual hearing aid program providing less gain for patients with sound tolerance issues may also be used to reduce the amplification of ambient sounds to prevent distress from sudden environmental sounds [5]. The use of additional technology now available in hearing aids may also be useful, such as setting up a patient’s hearing aids with their phone app to give them increased fine control of their hearing aid settings for their comfort. It is possible that for some patients knowing they have the control if they need it is enough to ease anxiety. Audiologists should also be prepared to follow-up closely with patients who have a history of trauma as these patients are often in historically marginalized groups, such as LGBTQ+ people, and may need closer follow-up to avoid losing them to follow-up [6]. Becoming trauma informed is important both formally and informally. The field of audiology needs training-specific hearing healthcare as well as informal conversations, articles, and actions that clinicians can implement to ensure the provision of high-quality care.

Contemporary hearing and balance testing may provoke trauma responses from claustrophobic environments, invasive testing, sudden loud sounds, or other triggers specific to an individual. Obtaining valid testing is a cornerstone of audiology, but these results cannot be obtained at the expense of re-traumatizing a patient. To avoid this possibility modifications can be made to audiologic tests to make them less distressing for patients with a history of trauma.

### 4.1. Claustrophobia in Testing Booths

Often past traumas lead to phobias. Phobias can begin at any age and may persist for years, so they may be present in any population seen in the audiology clinic. In addition, phobias are more common for women [32]. This is pertinent for audiology since woman show a higher prevalence of conditions such as otosclerosis and vestibular migraines, potentially leading to a particularly high morbidity of phobias in patients coming to audiologists with hearing or balance complaints [32]. One common phobia that may be triggered from past trauma during hearing or balance testing is claustrophobia.

Hearing testing in sound booths may trigger claustrophobia, particularly for veterans with PTSD who do may not tolerate being enclosed without an easy escape [5,33]. Some scholars have discussed how trying to “power through” claustrophobia despite patient distress can influence pure tone threshold testing, reducing test accuracy and causing results that may be consistent with nonorganic hearing loss [34]. This negates the clinicians’ goal of obtaining valid test results. Though some authors have proposed non-booth testing as an alternative, this could require specialized equipment that may not be easily available to clinicians and may not meet sound-isolating standards for hearing testing [33].

The American National Standards Institute (ANSI) is the organization that administers and coordinates voluntary standards in the United States including the standard for ambient noise in hearing-testing environments. While the standard itself is not law, it may be referenced by state or federal laws to ensure a common practice for obtaining test results. In the case of a patient where the typical test room could impact the validity of testing due to claustrophobia, clinicians should be flexible and consider satisfactory alternatives. Some possibilities for patients struggling with claustrophobia could be testing outside of a confining space that meet permissible noise levels, monitoring noise levels throughout testing, and documenting if noise levels exceed the standard. It should also be noted that the 1999 standard sets the maximum permissible ambient noise level to ensure appropriate testing at 0 dB HL. If testing was only going to be conducted down to 20 dB HL, then 20 dB can be added to the frequency for the transducers used [35]. Adding 20 dB to the allowable ambient noise levels could make it possible for clinicians to complete testing in a space within their clinic that is not a space that could trigger an individual with claustrophobia. Other options to complete testing could be orienting a patient to face the door of the booth while keeping the door open or closed or reducing clutter in the sound booth to make the area feel more spacious. In this situation being flexible and creative will allow for testing to be completed that provides valid results without re-traumatizing the patient.

### 4.2. Speech Testing

Clinicians should remember that subjective experience, not the objective invasiveness of procedures, determines the severity of potentially traumatic medical experiences [6]. One common evaluation that needs to be considered is speech testing. In these evaluations, words or sentences not only need to be heard by the patient but need to be repeated. Common word lists used during comprehensive evaluations contain words that may bring up past traumas for patients. Words such as “kill”, “death”, and “gun” are often avoided by clinicians during speech testing. For those administering the testing, the frequency of hearing these words in the testing context can neutralize the word for that individual. It is important to consider the impact of these words and either limit their use or possibly use different lists to ensure the validity of testing and reduce the negative impact on patients.

### 4.3. Acoustic Stapedial Reflexes and UCL Testing

Loud sudden sounds such as in UCL or acoustic reflex testing, can be distressing and cause startle responses that reduce testing accuracy among people with PTSD [5]. Some authors in other fields have suggested abbreviating distressing medical testing to focus on the clinical questions of most interest as efficiently as possible—omitting acoustic reflex or UCL testing when this testing would not change the diagnosis or recommendations may be preferrable [36]. If results from this testing are deemed necessary, the clinician can consider shortening the test to obtain the minimum results needed to add to a clinician’s decision-making. Discussion about the process of obtaining results is important for this testing and the patient should have the control to discontinue testing at any time.

### 4.4. Vestibular Testing

Vestibular testing is another source of potential distress for people with a history of trauma. The dark testing environment typically used in a rotary chair and VNG may be scary, particularly for children, and may be distressing for people with claustrophobia [37,38]. Goggles similar to those used in VNG testing have been shown to cause claustrophobia, and some authors have recommended ENG as a less claustrophobia-inducing alternative to VNG [39,40]. Caloric testing is significantly uncomfortable for most patients, so using a screening procedure like the MWST (monothermal warm screening test), can be used to reduce the number of caloric conditions a patient experiences during an appointment [41].

## 5. Conclusions

Audiology clinics see individuals across the lifespan. Any of these patients can have a history of ACEs and other trauma. These experiences have been associated with changes in hearing and balance disorders, with social determinants of hearing health, such as unemployment, and with the ability of patients to participate in audiologic testing and treatment. TIC strategies can be used by audiologists to identify, assess, and respond to trauma in their patients while avoiding retraumatizing patients during hearing or balance testing and treatment. TIC requires training and thoughtful implementation, so clinicians are encouraged to pursue TIC training and implement these strategies into their practice.

## Data Availability

No new data were created or analyzed in this study.
